# Sub-Lethal Irradiation of Human Colorectal Tumor Cells Imparts Enhanced and Sustained Susceptibility to Multiple Death Receptor Signaling Pathways

**DOI:** 10.1371/journal.pone.0031762

**Published:** 2012-02-28

**Authors:** Victoria Ifeadi, Charlie Garnett-Benson

**Affiliations:** Department of Biology, Georgia State University, Atlanta, Georgia, United States of America; Wayne State University School of Medicine, United States of America

## Abstract

**Background:**

Death receptors (DR) of the TNF family function as anti-tumor immune effector molecules. Tumor cells, however, often exhibit DR-signaling resistance. Previous studies indicate that radiation can modify gene expression within tumor cells and increase tumor cell sensitivity to immune attack. The aim of this study is to investigate the synergistic effect of sub-lethal doses of ionizing radiation in sensitizing colorectal carcinoma cells to death receptor-mediated apoptosis.

**Methodology/Principal Findings:**

The ability of radiation to modulate the expression of multiple death receptors (Fas/CD95, TRAILR1/DR4, TRAILR2/DR5, TNF-R1 and LTβR) was examined in colorectal tumor cells. The functional significance of sub-lethal doses of radiation in enhancing tumor cell susceptibility to DR-induced apoptosis was determined by in vitro functional sensitivity assays. The longevity of these changes and the underlying molecular mechanism of irradiation in sensitizing diverse colorectal carcinoma cells to death receptor-mediated apoptosis were also examined. We found that radiation increased surface expression of Fas, DR4 and DR5 but not LTβR or TNF-R1 in these cells. Increased expression of DRs was observed 2 days post-irradiation and remained elevated 7-days post irradiation. Sub-lethal tumor cell irradiation alone exhibited minimal cell death, but effectively sensitized three of three colorectal carcinoma cells to both TRAIL and Fas-induced apoptosis, but not LTβR-induced death. Furthermore, radiation-enhanced Fas and TRAIL-induced cell death lasted as long as 5-days post-irradiation. Specific analysis of intracellular sensitizers to apoptosis indicated that while radiation did reduce Bcl-X_L_ and c-FLIP protein expression, this reduction did not correlate with the radiation-enhanced sensitivity to Fas and/or TRAIL mediated apoptosis among the three cell types.

**Conclusions/Significance:**

Irradiation of tumor cells can overcome Fas and TRAIL resistance that is long lasting. Overall, results of these investigations suggest that non-lethal doses of radiation can be used to make human tumors more amenable to attack by anti-tumor effector molecules and cells.

## Introduction

Ionizing radiation (IR) has been administered clinically for the treatment of a wide range of human cancers for more than 100 years. Currently, it is the standard of care for many cancers, including colorectal cancer [1]–[3]. As a definitive therapy, radiation therapy (RT) has been used for the local control of tumor growth. Used in this manner, RT fails to control disseminated metastatic disease [4], which remains the primary cause of mortality of colorectal cancer patients [5], [6]. Moreover, many tumors develop resistance to death induction by radiation.

To overcome this barrier research and clinical trials have demonstrated that combining RT with other treatments is often more effective than RT alone [7], [8]. In this regard numerous studies indicate that IR has immuno-stimulatory properties and can enhance immune responses to tumor cells [9]–[16] and there is a wide array of immunotherapy strategies under clinical investigation in combination with RT [17]. The host immune system functions to suppress tumor cell growth in a process called tumor immunosurveillance [18] and important anti-tumor agents under consideration include both immune cells and immune effector molecules [19]–[23]. Many of these clinical investigations utilize RT as an adjuvant to such novel, immune-based therapies [13], [24]–[26]. While some of these studies reported enhanced immunological responses, none of the studies using RT as an adjuvant to immune-based therapy have reported significant reduction in tumor burden following therapy. Thus, better defining the molecular details of enhanced immune modulation by IR is critical to optimizing this strategy.

Death receptors of the tumor necrosis factor receptor (TNF) superfamily such as Fas receptor (Apo1/CD95), death receptor 4/TNF-Related apoptosis-Inducing ligand receptor 1 (DR4/TRAIL-R1), DR5 (TRAIL-R2), TNF-R1, and lymphotoxin-beta receptor (LTβR), are capable of inducing apoptotic signals into tumor cells following ligation with cognate death ligands from anti-tumor immune cells [27]–[32]. However, tumor cells can develop resistance to elimination by immune cells in a process termed immunoediting [33]. Numerous studies have suggested that inhibition of apoptotic death signaling pathways is a major mechanism of escape from immune cell elimination, as both cytolytic T-cells (CTL) and natural killer (NK) cells kill target cells using these mechanisms. Interestingly, we have shown that radiation can enhance or induce sensitivity to killing of tumor cells by CTLs [34], [35]. Our study explores the impact of sub-lethal doses of ionizing radiation on multiple death receptor pathways that could enhance productive interactions between cytolytic immune cells and tumor cells.

TNF-related apoptosis-inducing ligand (TRAIL) is expressed on numerous immune effector cells, including anti-tumor CTLs and NK cells [28]. Ligation of TRAIL with DR4 or DR5 on tumor cells induces the extrinsic apoptotic signal pathway, resulting in death of target cells. Several investigators have pursued soluble recombinant TRAIL, or agonistic antibodies to TRAIL-R, as anticancer therapeutics [36]–[38]; though, many tumor cells exhibit resistance to TRAIL killing [39]–[42]. Recent investigations have sought ways to overcome this TRAIL-resistant phenotype using a variety of therapeutic agents [41], [43]–[45]. Although TRAIL and Fas induce apoptosis by highly similar mechanisms, Fas agonists have not been pursued as aggressively as TRAIL agonists due to early observations of acute/lethal toxicity [46]. Utilization of these pathways by anti-tumor effector CTLs or NK cells is an attractive alternative to the administration of soluble agonists. This approach also offers the potential for long-lasting circulation of effector/memory CTLs in comparison to systemic drugs.

Colorectal tumor cells develop resistance to apoptosis signaling through Fas [47], [48], and Fas sensitivity decreases as tumor cells progress to advanced stage and metastatic disease phenotypes [49]. We and others have shown that radiation can modulate expression of death receptors in tumor cells, and it has been suggested that up-regulation of Fas following irradiation plays a significant role in enhanced killing of diverse tumor cells by CTL [50]–[53]. An in vivo murine radiation-immunotherapy study reported that implanted tumors expressing dominant negative Fas were not killed following irradiation, while functional Fas expressing tumors were killed [50]. This mechanistic observation has not borne out, however, in in vitro studies utilizing human carcinomas [34], [35], [54]. No correlation was found between increased levels of surface Fas expression and enhanced killing by antigen specific CTLs following irradiation [34]. In fact, results demonstrate that colorectal cells expressing surface Fas that is non-functional (SW620), still exhibit enhanced lysis by CTL following irradiation, indicating that enhanced susceptibility is not exclusively Fas-dependent. Radiation has also been reported to increase expression of TRAIL receptors on a variety of tumor cell types [44], [55]–[58]. Furthermore, IR has been shown to modulate sensitivity to TRAIL-induced apoptosis [59]. Thus, radiation has the potential to enhance apoptotic responsiveness through alternate pathways.

In this study, we explore the hypothesis that colorectal carcinoma cells can be modulated to become more sensitive to killing through death receptors utilized by cytotoxic immune cells, following exposure to sub-lethal irradiation. For these studies we sought to further characterize the impact of sub-lethal doses of radiation on death receptor (DR) expression, and to explore the functional impact of radiation on cell death induced through these pathways. We also sought to further explore the impact of radiation across multiple doses as there are limited studies, restricted to a single dose of radiation, specifically evaluating colorectal cells. This will help drive the design of combination radiation immunotherapy strategies. Specifically, we examined: a) the effects of ionizing radiation, at sublethal doses, on the potential modulation of DR expression, b) the functional consequences of radiation on DR killing, c) the longevity of radiation induced changes, and d) the role of intracellular sensitizers, such as bcl-X_L_ and c-FLIP (an integral element of apoptotic cell death pathways), in the potential regulation of colon carcinoma apoptotic outcome in combination treated cells.

To our knowledge, this is the first study to: a) simultaneously evaluate the effect of tumor cell irradiation on multiple common death receptors (Fas, TRAILR, LTβR and TNFR1), as well as the intracellular sensitizers to death in numerous colorectal carcinoma cells lines in a single study, b) evaluate multiple sub-lethal doses, and c) evaluate the longevity or sustainability of these changes.

## Results

### Sub-lethally irradiated colorectal tumor cells continue to proliferate

**Figure 1:**
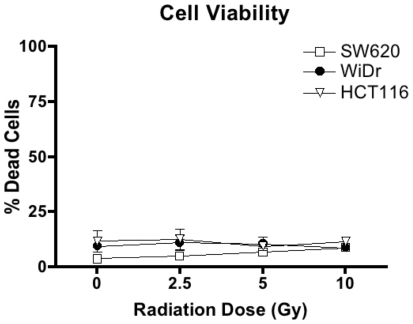
Tumor cells remain viable after 2.5, 5 and 10 Gy of radiation. A. SW620 (squares), WiDr (circles) and HCT116 (triangles) cells were irradiated with 2.5, 5 or 10 Gy of ionizing radiation. Irradiation and non-irradiated cells (0 Gy) were cultured for 72 h. Adherent cells were subsequently harvested and cell death was analyzed by 7AAD staining and flow cytometric analysis. Experiment was repeated 3 times with similar results.

As tumor cells are known to become radio-resistant, we wanted to separate the death inducing activity of radiation from it's ability to functionally and phenotypically alter tumor cells. To investigate the influence of sub-lethal doses of ionizing radiation on colorectal tumor cells we utilized 2.5, 5, and 10 Gy of ionizing radiation. A minimal increase in dead cells was observed. Treatment of SW620 cells with 10 Gy of radiation resulted in 8.65% of dead cells, as measured by 7AAD staining (**Fig. 1**), compared with non-irradiated cells that contained 5.48% dead cells. The maximum amount of cell death observed among the cell lines was 12% in HCT116 cells irradiated with 10 Gy, compared to 7% cell death in cells receiving 0 Gy. These cells were the most sensitive to radiation. WiDr cells displayed the least amount of variation in cell death as non irradiated (0 Gy), 2.5 Gy and 5 Gy samples contained 8% dead cells, and WiDr cells irradiated with 10 Gy contained 9% dead cells. All other cell lines at all other doses resulted in less than 10% dead cells. This was repeated several times with no significant increase in cell death observed among the irradiated colorectal tumor cells. Furthermore, there was no decrease in viability as measured by trypan blue uptake on irradiated SW620, HCT116, and WiDr cells (data not shown), nor was there an increase in early apoptotic cells as measured by Annexin-V staining (data not shown). Thus, the levels of irradiation utilized in these experiments were considered to be sub-lethal in these cell lines.

**Figure 2:**
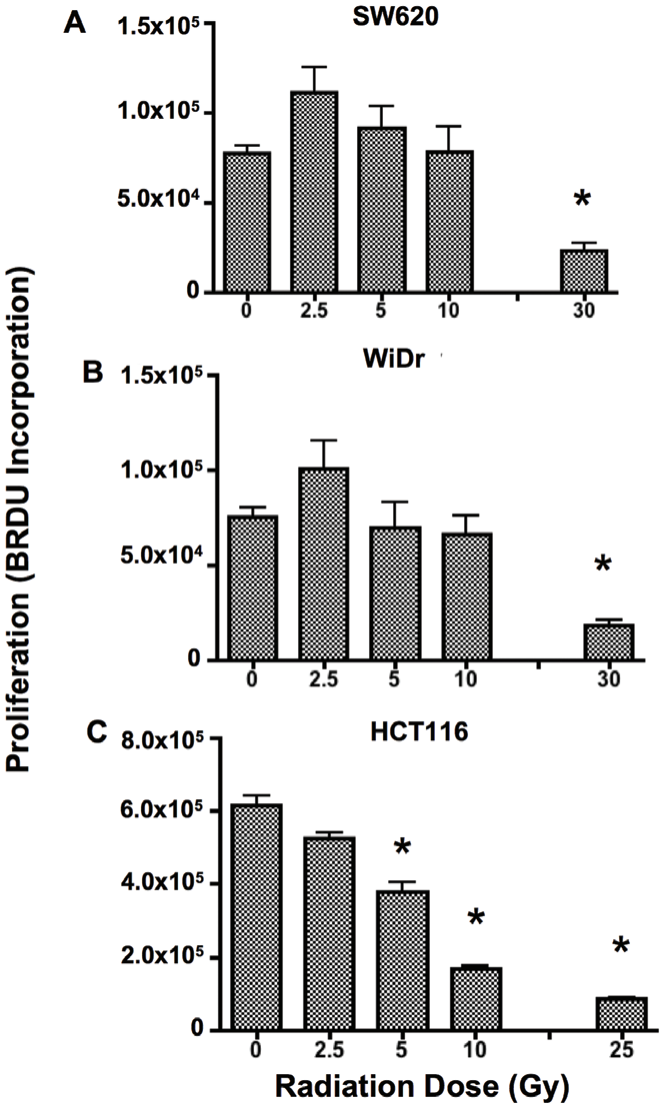
Tumor cells continue to proliferate following sub-lethal doses of radiation. Proliferation was measured 72 h after receiving 0, 2.5, 5, or 10 Gy of radiation by measuring BRDU incorporation. Cells receiving 25 or 30 Gy of irradiation were used as a positive control for inhibition of proliferation. A. Proliferation of SW620 cells. B. Proliferation of WiDr cells. C. Proliferation of HCT116 cells. Samples were done in triplicate at each dose. Experiment was repeated 3 times with similar results.

The impact of radiation on tumor cells is a balance between cell death and cell proliferation. Therefore, we investigated the impact of these sub-lethal doses of radiation on tumor cell proliferation by measuring BRDU incorporation. 2.5 Gy of irradiation did not significantly decrease proliferation of any of the three cell lines used in this study (**Fig. 2**). Proliferation of SW620 and WiDr cells was also not reduced following 5 or 10 Gy of radiation (**Fig. 2A & 2B**). Proliferation could, however, be significantly reduced by administration of 30 Gy of radiation to both SW620 and WiDr cells. In contrast, proliferation of HCT116 cells was impacted significantly by radiation both at 5 Gy and 10 Gy (**Fig. 2C**). Proliferation was reduced by 30% at 5 Gy and reduced by 60% following 10 Gy. Importantly proliferation of HCT116 was not completely eliminated (**Fig. 2C**), especially when compared to 25 Gy of radiation. Together these data indicate that viable and proliferating cells remain after exposure to the doses of radiation used in our studies.

### Sub-lethal irradiation of colorectal tumor cells can enhance sensitivity to killing through Fas receptor that is sustained

**Figure 3:**
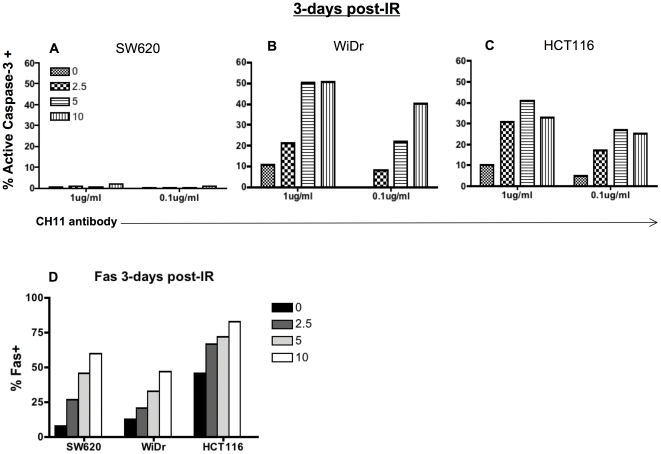
Functional enhancement of Fas receptor pathway in sub-lethally irradiated colorectal tumor cells. Human tumor cells were mock-irradiated (0 Gy) or irradiated with 2.5, 5 or 10 Gy and re -cultured for 72 h (3-days). Cells were harvested and incubated with the indicated concentrations of Fas crosslinking antibody CH11 or IgM isotype control antibody for 3 h. A. Non-functional Fas receptor signaling in SW620 cells 72 h post-IR. B. Functional Fas receptor signaling in WiDr cells 72 h post-IR. C. Functional Fas receptor signaling in HCT116 cells 72 h post-IR. Experiment was repeated 3 times with similar results. D. Surface Fas levels, as determined by flow cytometry of stained cells 72 h post-IR.

Fas ligand (FasL) expressing CTL and NK cells can kill tumor cells expressing the Fas receptor [28]. Radiation has been shown to increase surface expression of Fas, however, numerous colorectal cell lines, including HCT116 [27] and SW620 cells [49], have been reported to be resistant to killing through Fas. Moreover, they have also been shown to be resistant to radiation-mediated apoptosis at 2–6 Gy of radiation. Interestingly, simultaneous administration of 10 Gy of radiation and a novel Fas crosslinking antibody, APO010, to HCT116 has recently been reported to result in enhanced DNA fragmentation [60]. Differences reported on the sensitivity of HCT116 cells to Fas signaling appear to be due to differences in cell sensitivity to anti-Fas mAb vs FasL recombinant protein. We wanted to explore the impact of combining these two treatments on apoptosis induced through the Fas receptor in numerous colorectal cell lines. We also wanted to further explore the impact of radiation dose. To this end, we treated SW620, HCT116, and WiDr cells with 2.5, 5, and 10 Gy of irradiation. We initiated Fas-mediated death 72 h post-irradiation using the anti-Fas crosslinking antibody CH11. Fas-mediated apoptosis was measured by active caspase-3 detection. SW620 cells have been reported to be resistant to killing through Fas [49], and radiation did not change susceptibility to Fas death in SW620 cells (**Fig. 3A**). In contrast, treatment of both HCT116 cells and WiDr cells with radiation resulted in enhanced Fas-mediated apoptosis (**Fig. 3B & 3C**). This was detected when death was induced using both high (1 µg/ml) and low (0.1 µg/ml) concentrations of CH11 antibody. Enhanced cell death correlated well with increasing doses of radiation. In the absence of CH11 antibody, active caspase-3 levels were less than 5% in all cell lines, confirming that the doses of radiation used here can be considered sub-lethal in these cells (data not shown).

The radiation-induced enhanced susceptibility to Fas did not seem to be dependent on the magnitude of change observed in surface levels of Fas detected by flow cytometry. 10 Gy of radiation increased Fas expression to 60% in SW620 (**Fig. 3D**), which had no susceptibility to Fas-induced death at any of the doses of radiation. We found that 83% of HCT116 cells expressed Fas upon 10 Gy radiation, as compared to 47% of WiDr cells receiving the same dose (**Fig. 3D**). While HCT116 cells demonstrated the largest increase in Fas expression post-irradiation, WiDr cells demonstrated the largest increase on Fas-induced apoptosis, reaching 50% apoptotic cells upon treatment with 1 µg/ml of CH11 (compared to approximately 40% apoptotic cells observed in HCT116). Therefore increased surface expression of Fas could not be used as the predictor for sensitivity to Fas-mediated cell death.

**Figure 4:**
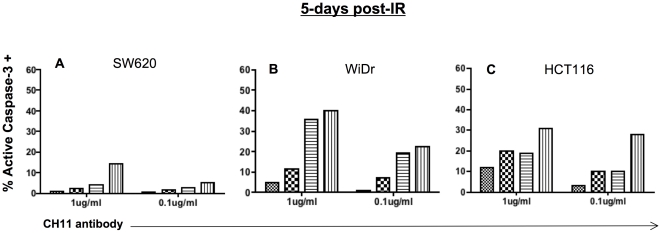
Functional enhancement of Fas receptor pathway in sub-lethally irradiated colorectal tumor cells is sustained. Human tumor cells were mock-irradiated (0 Gy) or irradiated with 2.5, 5 or 10 Gy and re -cultured for 120 h (5-days). Cells were harvested and incubated with the indicated concentrations of Fas crosslinking antibody CH11 or IgM isotype control antibody for 3 h. A. Functional Fas receptor signaling in SW620 cells 120 h post-IR. B. Functional Fas receptor signaling in WiDr cells 120 h post-IR. C. Functional Fas receptor in HCT116 cells 120 h post-IR. Experiment was repeated 3 times with same results.

Both WiDr and HCT116 cells exhibited enhanced susceptibility to Fas-induced cell death 72 h after exposure to radiation. It is unclear how long radiation is able to sensitize tumor cells to killing through Fas, and we next evaluated if this increased sensitivity to killing through Fas would remain so at a later time post-irradiation. To evaluate this possibility, we sub-lethally irradiated cells and waited five days before triggering death through the Fas receptor. Interestingly, both cell types remained more sensitive to Fas-induced killing if exposed to radiation first (**Fig. 4B & 4C**). Both WiDr cells and HCT116 cells show nearly a 10% reduction in the level of apoptotic cells as was observed at the earlier time post-irradiation using 1 µg/ml of antibody. Importantly, both cell lines remained sensitive even when the lower dose of CH11 antibody was used. Surprisingly, SW620 cells demonstrated some sensitivity to killing through the Fas receptor at day five that was not observed 3 days post irradiation (**Fig. 4A**). Approximately 20% of SW620 cells contained active caspase-3 following the combination of 10 Gy and 1 µg/ml of CH11 and this result was observed each time the experiment was conducted. These data suggest that low doses of radiation can sensitize colorectal tumor cells to killing through the Fas receptor that would not be sensitive without radiation pre-treatment and that this effect lasts as long as 5 days post-IR.

### Radiation increases expression of TRAIL receptors, but not of TNFR1 or LTβR, in colorectal tumor cells

**Figure 5:**
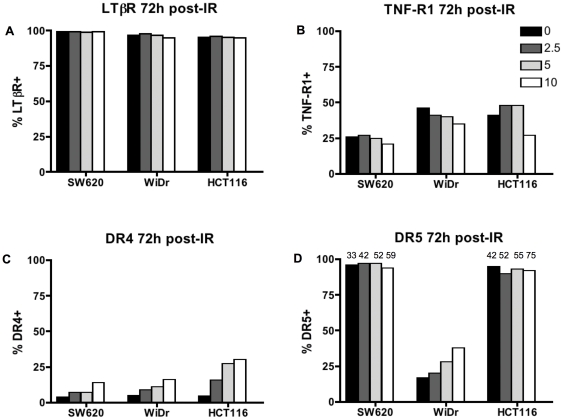
Radiation can modulate surface expression of some but not all TNF family death receptors in colorectal tumor cell lines. SW620, WiDr and HCT116 cells received 0 (black bar), 2.5 (dark grey bar), 5 (light gray bar) and 10 Gy (white bar) of radiation. Cells were re-cultured for 72 h and then analyzed by flow cytometry for surface (A) LTβR, (B) TNF-R1, (C) DR4, and (D) DR5 surface expression (inset; MFI of DR5 expression is shown for SW620 and HCT116 cells). Percent of cells expressing each death receptor is graphed. Cells stained with a fluorescently-labeled isotype control antibodies were negative (not shown). Staining was repeated 3 times with similar results.

Numerous members of the TNF family of receptors are capable of inducing death of tumor cells. We next wanted to evaluate if sub-lethal doses of radiation were capable of modulating expression of other death receptors. Lymphotoxin-α (LTα) and LTβ can be expressed by T-cells and function as a mediator of tumor cell death through the LTβR [61], [62]. Tumor cell death can also be induced by ligation of this receptor by CD258/LIGHT (for homologous to lymphotoxins, exhibits inducible expression, and competes with HSV glycoprotein D for HVEM, a receptor expressed by T lymphocytes) [63]. There are no reports on the impact of radiation on tumor cell expressed LTβR. We examined surface expression of LTβR on SW620, WiDr, and HCT116 cells three days post-irradiation (**Fig. 5A**). In contrast to the up-regulation observed in expression of Fas receptor, we observed no change in the percent of cells expressing LTβR 72 h post-irradiation with 2.5, 5 or 10 Gy. In fact, each of the cell lines evaluated expressed LTβR on greater than 95% of the cells. We also did not observe an increase in the amount of this receptor on the cell surface as determined by mean fluorescence intensity (data not shown). Interestingly, our data reveal that all of the colorectal tumor cells examined in these studies express high levels of this receptor.

Kimura (2000) et al. demonstrated enhanced activation of apoptotic effector molecules following irradiation and treatment of prostate cancer cells with TNF-α [64]and modulation of TNF-R1 has been observed in breast cancer cells following IR [56]. In contrast, the three colorectal cell lines evaluated here exhibited no change in surface expression of TNF-R1 72 h post-irradiation with 2.5 or 5 Gy (**Fig. 5B**). We did observed a small reduction in expression following 10 Gy IR in all three cell lines. We also evaluated cells one day and four days post-irradiation and observed similar changes (data not shown).

Radiation has been reported to modulate protein expression of TRAIL receptors in prostate [44], [64], breast [56], and gastric cancer [57], [58] cells. Lastly, we wanted to evaluate the surface expression of the TRAIL receptors DR4/TRAILR1 and DR5/TRAILR2 in our three colorectal cell lines following exposure to sub-lethal doses of radiation. Surface expression of DR4 was less than 10% in all three cell lines prior to irradiation (**Fig. 5C**). Three days after irradiation, each of the three cell lines demonstrated increased surface expression in a dose dependent manner. HCT116 cell showed the largest increase (<5% in 0 Gy versus >35% in 10 Gy treated cells). A different pattern of expression was observed for DR5 expression. WiDr cells were the only cell line that expressed low levels of DR5 (15%) on the cell surface before irradiation (0 Gy) (**Fig. 5D**). A dose-dependent increase in surface DR5 expression was observed in these cells 72 h after radiation. In contrast, both SW620 and HCT116 cells expressed high levels (>95%) of DR5 in the absence of radiation. However, radiation did induce an increase in the density of DR5 on the surface of these cells in a dose-dependent manner as measured by mean fluorescence intensity (**Fig. 5D**; inset MFI numbers). Overall, these data reveal that sub-lethal doses of IR induce increased expression of some death receptors (Fas, DR4 and DR5) but not all TNF related death receptors (LTβR and TNF-R1) in colorectal tumor cells.

**Figure 6:**
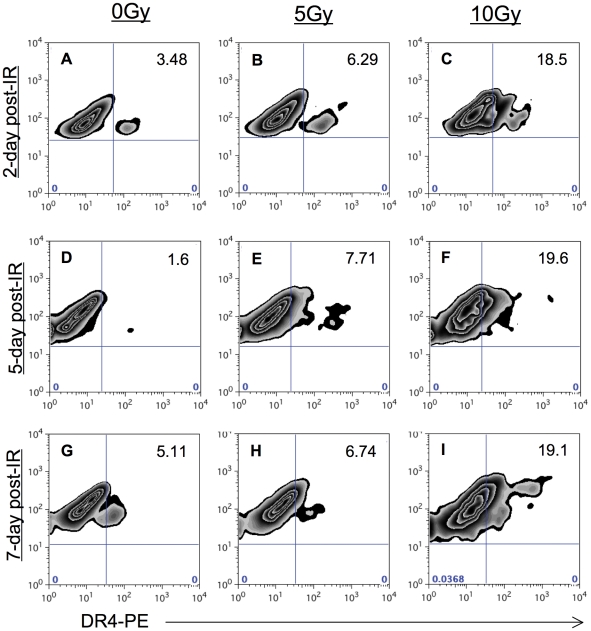
Radiation-induced increase in DR4 expression is sustained for up to a week. HCT116 cells were irradiated with 0, 5 or 10 Gy irradiation. Cells were re-cultured, harvested and subsequently stained for surface DR4 after 2 days (A–C), 4 days (D–F), and 7 days (G–I). Each experiment was repeated twice with similar results. Cells stained with a PE-labeled isotype control antibody were below 5% (not shown).

To determine how early radiation induced such changes and how long they may last, we evaluated expression of DR4 at two, four, and seven days post-irradiation. Radiation-induced up-regulation was detectable as early as 48 h (**Fig. 6A–6C**) and remained detectable as long as four (**Fig. 6D** **–** **5F**) and seven days (**Fig. 6G–6I**) after radiation of HCT116 cells. Furthermore, increased expression of DR4 was still detectable in both SW620 and WiDr cells seven days post-irradiation (data not shown). Similarly, increase expression of DR5 also remained detectable seven days after radiation (data not shown).

### Sub-lethal irradiation of colorectal tumor cells can enhance sensitivity to killing through TRAIL receptors that is sustained

**Figure 7:**
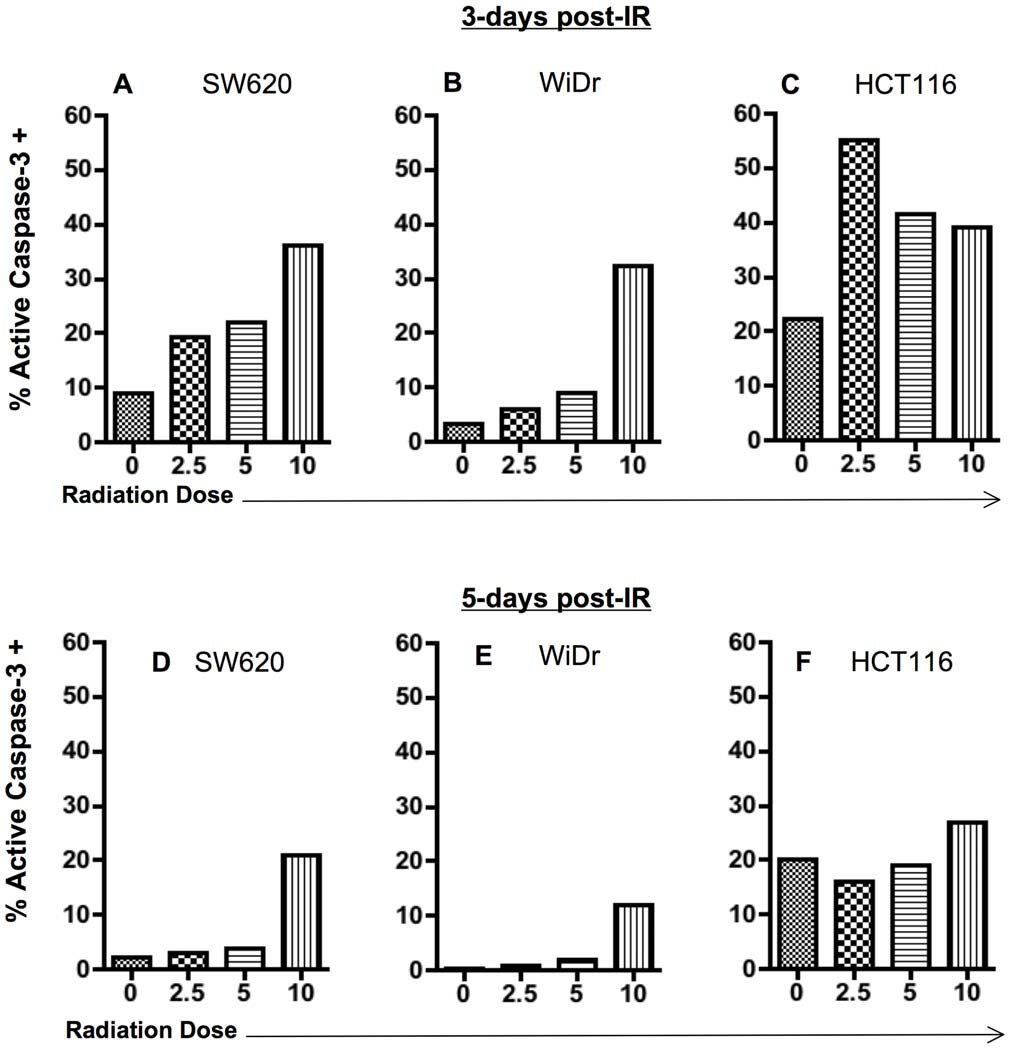
Functional enhancement of TRAIL receptor pathway in sub-lethally irradiated colorectal tumor cells is sustained. Human tumor cells were mock-irradiated (0 Gy) or irradiated with 2.5, 5 or 10 Gy and re-cultured for the indicated times. Cells were harvested and incubated with the indicated concentrations of recombinant TRAIL protein or media for 3 h. Functional TRAIL receptor signaling in (A) SW620, (B) WiDr and (C) HCT116 cells 72 h post-IR (3-days). SW620 and WiDr cells were incubated with 100 ng/mL of recombinant protein and HCT116 cells were incubated with 25 ng/mL of protein. Experiments were repeated 3 times with similar results. Functional TRAIL receptor signaling in (D) SW620, (E) WiDr, and (F) HCT116 cells 120 h (5-days) post-IR. Experiments were repeated 2 times with similar results.

Radiation has previously been reported to very mildly modulate sensitivity to TRAIL-induced apoptosis [59] in one of two colorectal tumor cells lines (HCT116 cells and Colo205 cells). However, these studies utilized independent agonists for DR4 (mapatumumab) and DR5 (lexatumumab) in combination with 5 Gy of radiation. There was a very small enhancement in apoptosis through DR5 when used in combination with 5 Gy radiation in HCT116 cells (25% dead cells with DR5 agonist alone versus 40% dead cells with IR+lexatumumab), and no enhancement in killing through DR4. For these studies, irradiation was administered immediately before administration of antibodies and apoptosis was detected 36 h after treatment. Similar studies have evaluated co-treatment strategies on other tumor cells derived from solid cancers. We wanted to more specifically investigate pre-treatment with irradiation on subsequent killing of colorectal tumor cells through death receptors. In addition, we sought to evaluate whether radiation could enhance killing through TRAIL receptors, several days later, using the soluble recombinant protein capable of stimulating apoptosis through both DR4 and DR5. SW620, HCT116, and WiDr cells were treated with 2.5, 5, and 10 Gy of irradiation. TRAIL receptor-mediated death was initiated using recombinant TRAIL protein 72 h post-irradiation and receptor-mediated apoptosis was measured by active caspase-3 detection. We observed increased susceptibility to apoptosis induction by TRAIL signaling in each of the three cell lines evaluated in this study (**Fig. 7A–C**). Interestingly, SW620 cells, which were not rendered sensitive to Fas killing by radiation, were more sensitive to cell death via TRAIL following all doses of radiation (**Fig. 7A**). This was observed even with radiation doses as low as 2.5 Gy. Cell death was of higher magnitude when 100 ng/ml of TRAIL were used, as compared to the lower dose of 25 ng/ml (data not shown). A similar enhancement in TRAIL mediated apoptosis was also observed with WiDr cells (**Fig. 7B**) and HCT116 cells (**Fig. 7C**). HCT116 cells were sensitive to TRAIL-mediated apoptosis both with and without irradiation when 100 ng/ml of protein were used, and no increase in apoptosis was observed (data not shown). However, the impact of irradiation was observable using the lower dose of recombinant protein (25 ng/ml) (**Fig. 7C**). Irradiation of HCT116 cells with 5 Gy IR followed by TRAIL receptor activation three days later demonstrated almost twice as much apoptosis (>40%) when compared to non-irradiated cells (23%) (**Fig. 7C**). Overall, radiation enhanced TRAIL related death signaling in 3 of 3 colorectal tumor cell lines evaluated here.

We next wanted to investigate the longevity of this enhanced apoptosis observed through TRAIL by sub-lethal irradiation of tumor cells. We evaluated the sensitivity of irradiated cells to TRAIL receptor-mediated apoptosis five days after irradiation. Two of three lines (WiDr and SW620 cells) retained sensitivity to TRAIL following 10 Gy of radiation (**Fig. 7D–F**). The degree of sensitivity to TRAIL was reduced five days after radiation (**Fig. 7D& 7E**), though it was not completely abrogated and was still detectable. In contrast, HCT116 cells, which were already very sensitive to TRAIL-induced cell death, lost some radiation-enhanced sensitivity to TRAIL 5-days post irradiation as compared to the sensitivity 3-days post irradiation (**Fig. 7C & 7F**). Overall, these data suggest that sub-lethal doses of radiation can sensitize colorectal tumor cells to killing through the TRAIL receptors that would not be sensitive without radiation pre-treatment and that this effect can last as long as 5 days post-IR in some tumor cells.

### Sub-lethal irradiation of colorectal tumor cells does not enhance sensitivity to killing through LTβ receptors

**Figure 8:**
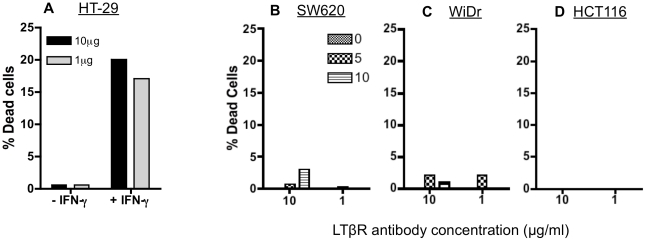
Cell death through the LTβR is not enhanced by sub-lethal irradiation of colorectal tumor cells. Cells were incubated on plates coated with 10 µg/ml or 1 µg/ml of anti- LTβR mAb (31G4D8) for 48–72 h. Cell viability was determined by 7-AAD uptake comparing cells treated with 31G4D8 versus cells cultured in medium alone and isotype control mAb. A. As a positive control HT-29 cells were incubated with 31G4D8 in conjunction with 50 units/ml IFN-γ (+IFN- γ), or without (-IFN- γ) as a negative control. B–D. Human tumor cells were mock-irradiated (0 Gy) or irradiated with 5 or 10 Gy and re-cultured for 72 h. SW620 (B), Widr (C) and HCT116 (D) cells were then harvested and incubated on plates coated with 10 µg/ml or 1 µg/ml of 31G4D8 for 48–72 h. Experiments were repeated 3 times with similar results.

Blocking LT*β*R with LT*β*R-specific neutralizing monoclonal antibody has been reported to decrease CTL cytotoxicity against tumor cells in vitro [32]. Furthermore, silencing LT*β*R using specific short hairpin RNA was reported to reduce the ability of perforin-deficient CTLs to induce tumor rejection in vivo. Each of the three cell lines evaluated in this study expressed higher levels of surface LT*β*R than Fas or TRAIL receptors. The agonistic anti-LT*β*R mAb, 31G4D8, has been reported to induce cell death in tumor cells in conjunction with IFN-γ [65]. As expected, HT-29 cells demonstrated enhanced LT*β*R-induced cell death upon treatment with IFN-γ as measured by 7AAD uptake by flow cytometry (**Fig. 8A**). No cell death was observed in cells treated with 10 µg/ml of 31G4D8 in the absence of IFN-γ, while death was detected in 20% of HT-29 cells upon IFN-γ treatment. Slightly less death (17%) was detected in cells treated with 1 µg/ml of 31G4D8. Not surprisingly, we observed no tumor cell death in SW620, HCT116, or WiDr cells following treatment with the LT*β*R mAb alone (**Fig. 8B–D**). To evaluate if radiation could sensitize cells to killing through this pathway, SW620, HCT116, and WiDr cells were treated with 5 and 10 Gy of irradiation. 72 h post-irradiation, LT*β*R-mediated death was initiated using anti-LT*β*R mAb. Under these conditions, tumor cell irradiation was not able to increase the susceptibility to cell death induced by LT*β*R signaling, and cell death remained below 3% in all cell lines (**Fig. 8B–D**). LT*β*R-mediated apoptosis was also measured by active caspase-3 detection as signaling through LT*β*R has been reported to enhance both caspase-independent and -dependent cell death [65]. We were unable to detect any active caspase-3 following LT*β*R activation in any of the cell lines evaluated, including the HT-29 cells treated with IFN-γ (data not shown).

### Radiation of colorectal tumor cells modulates intracellular sensitizers to apoptosis that do not correlate with radiation-enhanced susceptibility to Fas or TRAIL receptor death

**Figure 9:**
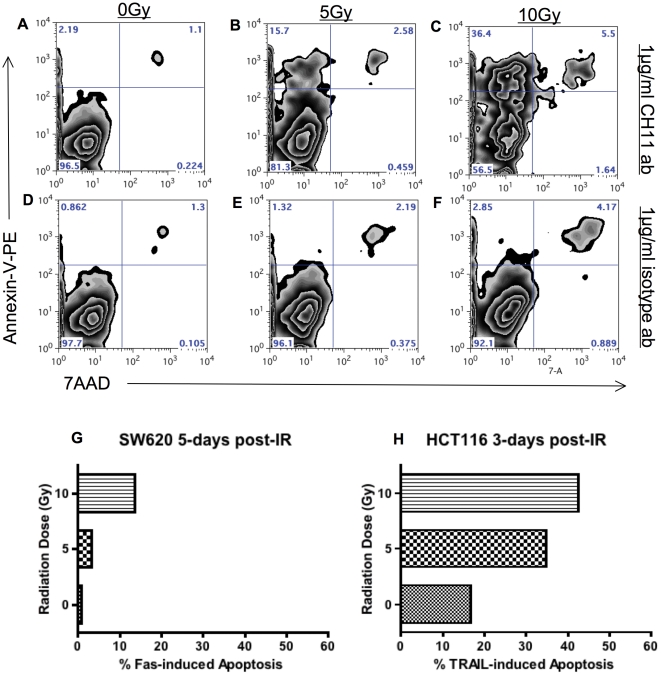
Enhanced sensitivity to receptor-mediated cell death by sub-lethal irradiation is apoptotic. Kinetics of dead and apoptotic cells were analyzed by annexin V–PE/7AAD staining of cells and flow cytometry. A–F. WiDr tumor cells were mock-irradiated (0 Gy) or irradiated with 5 or 10 Gy and re -cultured for 72 h. Cells were harvested and incubated with the indicated concentrations of Fas crosslinking antibody CH11 (A–C) or IgM isotype control antibody (D–F) for 5 h. Percent of apoptotic cells are represented in the upper left quadrant as Annexin-V^+^ and 7AAD^−^. B. Induction of apoptosis (Annexin-V^+^ and 7AAD^−^) following Fas receptor signaling in SW620 cells 120 h post-IR. Cells were treated with 1 µg/ml of CH11 mAb for 5 h. C. Induction of apoptosis (Annexin-V^+^ and 7AAD^−^) following TRAIL receptor signaling in HCT116 cells 72 h post-IR. Cells were incubated with 100 ng/ml of recombinant TRAIL protein for 5 h. Experiment was repeated 3 times with similar results.

We detected increased production of active caspase-3 following Fas and TRAIL induced cell-death in irradiated tumor cells. To confirm that cells with increased detection of active caspase-3 were truly undergoing apoptosis, we evaluated cells for surface expression of phosphatidylserine (PS) as an indicator of early apoptotic cells. Figure 9 shows the number of early apoptotic cells (annexin-V^+^/7AAD^−^) in WiDr cells treated 72 h post-IR with the agonistic Fas antibody (CH11). 2.19% of non-irradiated cells are undergoing apoptosis upon Fas signal induction as compared to 15.7% and 36.4% in cells irradiated with 5 and 10 Gy respectively (**Fig. 9A–C**). Irradiated cells treated with an isotype control antibody contain less than 3% apoptotic cells regardless of whether they were irradiated or not. Importantly less than 6% of cells in all treatment groups were in late apoptosis or necrosis as defined by annexin-V^+^/7AAD^+^ in this short 5 h assay. SW620 cells also demonstrated increased apoptosis following Fas receptor triggering 5 days post-IR (**Fig. 9G**). Similarly, increased apoptosis was also detected in irradiated tumor cells undergoing TRAIL-induced death (**Fig. 9H**). Thus, death induced by IR pre-treatment followed by death receptor ligation is apoptotic.

**Figure 10:**
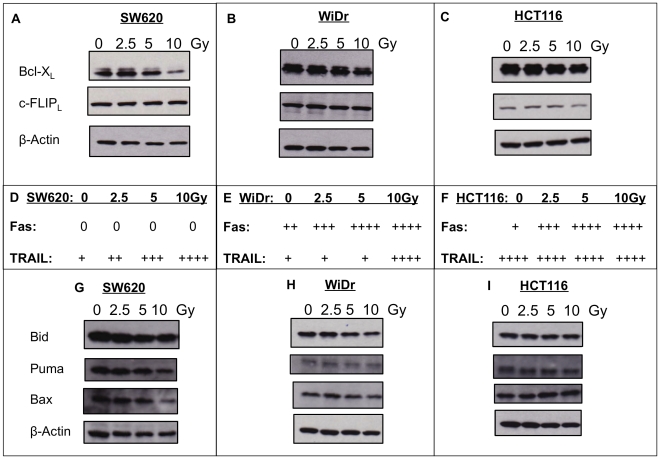
Changes in the expression of anti-apoptotic molecules following tumor cell irradiation. A–C. Expression of Bcl-X_L_ and c-FLIP was detected by western blot 72 h post-irradiation in SW620, WiDr, and HCT116 cells. β-actin was used as a loading control. D–E. Comparison of susceptibility to Fas (1 µg/mL) and TRAIL (100 ng/ml) death receptor induced apoptosis. Susceptibility to apoptosis is represented as: (0) denotes no apoptosis as <3%, (+) denotes 3–10%; (++) denotes 11–20%, (+++) denotes 21–30%, and (++++) denotes greater than 31% of cells positive for active caspase-3 in the functional death receptor assays. G–H. Expression of pro-apoptotic proteins Bid, Puma and Bax was detected by western blot 72 h post-irradiation in SW620, WiDr and HCT116 cells. β-actin was used as a loading control. Membranes were probed simultaneously and exposed to film for the same amount of time.

There are numerous well-characterized intracellular sensitizers to apoptotic death signals induced through death receptors. Molecules, such as Bcl-2, Bcl-X_L_, survivin, and c-FLIP are known to inhibit apoptosis [66], [67]. Bcl-X_L_ acts on the mitochondria of a cell by inhibiting the release of cytochrome c, which is needed to initiate apoptosis [39]. SW620 cells displayed marked decreased expression of Bcl-X_L_ upon 10 Gy radiation, as determined by immunoblotting (**Fig. 10A**). WiDr cells displayed mild reduction in Bcl-X_L_ protein at 10 Gy dose (**Fig. 10B**). In contrast, there was low to no reduction in Bcl-X_L_ expression in HCT116 cells (**Fig. 10C**). Surprisingly, SW620 cells had an increase in Bcl-X_L_ expression at low doses of IR (2.5 Gy) that was reproducibly observed in our experiments. β-actin was used as protein loading control for these experiments. cFLIP inhibits activation of caspase-8 needed for the execution of apoptosis [68], [69]. HCT116 cells were the only cells to respond to radiation by reproducibly decreasing expression of cFLIP following 10 Gy (**Fig. 10C**). No reduction in cFLIP protein was ever observed in either SW620 or WiDr cells (**Fig. 10A & 10B**). Thus IR was able to reduce expression of some anti-apoptotic proteins in colorectal tumor cells.

We next wanted to see if the enhanced functional killing through either Fas or TRAIL would correlate with the changes in anti-apoptotic proteins. Sensitivity to Fas and TRAIL induced apoptosis were compared with changes in expression of c-FLIP and Bcl-X_L_. SW620 displayed the greatest reduction in Bcl-X_L_ protein levels 72 h post-IR; however, SW620 cells are not rendered susceptible to Fas-induced apoptosis (**Fig. 10D**), while WiDr and HCT116 are very susceptible (**Fig. 10E & F**). There is no loss of cFLIP protein expression in either SW620 or WiDr cells, though Fas signaling results in apoptosis in WiDr cells (**Fig. 10E**), but not in SW620 cells. Indeed, WiDr cells are sensitive to both Fas and TRAIL killing and there is no major reduction in either Bcl-X_L_ or cFLIP. Most obviously, SW620 is sensitive to TRAIL killing, even at the dose of radiation that appears to increase expression of Bcl-X_L_ (2.5). Both WiDr and HCT116 cells are very sensitive to TRAIL killing following 10 Gy of IR. However, HCT116 exhibit a mild reduction in cFLIP protein expression at 10 Gy (and express the lowest overall levels), while WiDr cells show no change in cFLIP expression. Thus in our studies, radiation-induced changes in apoptotic inhibitors do not correlate with enhanced killing through Fas or TRAIL death receptors.

Enhanced susceptibility to death receptor signaling could also be the result of increased expression of necessary pro-apoptotic proteins. In some cell lines, death-receptor mediated activation of caspase-8 is known to simultaneously activate the mitochondrial pathway through production of Bid [70]. Increased expression of Bid has been reported to increase death receptor apoptosis [71]. We observed no increased expression of active Bid protein expression in the cells evaluated in this study at any of the doses of radiation evaluated (**Fig. 10G–H**). Bax, another pro-apoptotic protein, promotes death receptor mediated apoptosis via the mitochondria, and deletion of Bax has been reported to promote resistance to DR4/DR5 killing [72], [73]. No change in Bax expression was observed in WiDr or HCT116 cells following IR (**Fig. 10G–H**). In fact, we actually observed slightly reduced levels of Bax protein in SW620 cells treated with 10 Gy, although, these cells were still very susceptible to cell death through TRAIL. Puma is a p53-induced pro-apoptotic protein that promotes death by binding to pro-survival Bcl-2 family members and interfering with their interactions with pro-apoptotic Bak and Bax [74]. We observed mildly decreased expression of Puma in SW620 cells treated with 10 Gy and no change in expression in WiDr cells or HCT116 cells (**Fig. 10 G–H**). Thus enhanced death receptor signaling does not appear to be due to increased expression of pro-apoptotic proteins.

## Discussion

Colorectal tumor cells develop resistance to many apoptotic pathways, including Fas [47] and TRAIL [39]–[41]. Apoptosis decreases as tumor cells progress to advanced stage and metastatic disease phenotypes [49]. The aim of this study was to both characterize the expression of death receptors in colorectal cancer cells, and to seek to manipulate these levels with irradiation. Moreover, we wanted to evaluate the longevity of these changes. For these studies we used doses of radiation considered to be sub-lethal to separate out the direct cytotoxic effects of radiation from its ability to phenotypically modulate tumor cells (Fig. 1). Data from our study suggests that tumor cells that are not killed by radiation are still susceptible to death receptor initiated cell death. Cells receiving up to 10 Gy of irradiation continue to proliferate (Fig. 2), and this finding could be particularly relevant for radioresistant tumor cells. These data suggest that administration of radiation may still be relevant even in situations where tumor cells are not dying as a direct consequence of radiation. In addition, radiation can kill both malignant cells and non-malignant cells and can have significant dose-limiting toxicities, thereby making the use of lower radiation doses useful.

A murine in vivo model demonstrated elimination of tumor burden in CEA positive tumor cells in a CEA-Tg use model, using the combination of sub-lethal tumor cell irradiation and pox viral CEA expressing viral vectors [50]. This model showed that anti-tumor activity was dependent on Fas, as Fas expression was upregulated and anti-tumor activity was abrogated if cells expressing dominant-negative Fas were implanted instead. In contrast, experiments evaluating human tumor cell lines in vitro showed enhanced killing of irradiated tumor cells by CEA specific human CTLs that could not be correlated with surface Fas expression [34]. Furthermore, this enhanced killing by CTLs was exhibited in cells where the Fas pathway is non-functional. Here we show increased expression of surface Fas on three colorectal cell lines at doses as low as 2.5 Gy (Fig. 3D). Though surface Fas was increased in SW620 cells following radiation, these cells remain refractory to cell death initiated through the Fas receptor (Fig. 3A). These data suggest that expression of surface Fas receptor following therapy cannot be used to predict sensitivity to death through this receptor. This is further demonstrated by observations that the tumor cell line with the smallest increase in surface Fas (WiDr cells; Fig. 3D) had the most sensitivity to Fas-induced cell death signals (Fig. 3B). These results highlight important differences between observations made in murine models and those observed using human tissues. The focus of additional studies will be on the evaluation of the response to irradiation of tumor cells, isolated ex vivo, from human tumor samples.

We observed enhanced susceptibility to Fas death receptor signals in both WiDr cells and HCT116 cells (Fig. 3B–C) 3-days after sub-lethal tumor cell irradiation. This effect was observed when doses as low as 2.5 Gy were utilized, and was present at both high and low concentration of CH11 antibody. In contrast, Sheard et al. [48] reported radiation-induced up-regulation of Fas receptor expression that had little or no effect on responsiveness to CH11 antibody in HCT116 cells. Yet in their studies, protein synthesis was inhibited with cyclohexamide at the time of CH11 antibody administration. Thus it appears that enhanced susceptibility to Fas signaling is dependent on protein synthesis and modulated gene expression. Furthermore, Sheard et al. administered the antibody 4 h after 8 Gy irradiation. SW620 cells are resistant to Fas-induced cell death. Interestingly, we observed sensitization to Fas-induced death in a long-term culture (5 days) (Fig. 4A). These data suggesting that the timing of tumor cell irradiation and initiation of death receptor signaling may be important and clinicians may need to allow time for phenotypic modulation of tumor cells to achieve the maximum amount of cell death.

Targeting the TRAIL receptor is an attractive therapy for cancer, and numerous studies suggest that this pathway can be modulated by other therapies. Studies have shown increased sensitivity to TRAIL following IFNγ and TNFα treatment [41]. Others have reported that agents such as multikinase inhibitors, histone deacetylase inhibitors, and ceramide can modulate this signaling pathway [43], [45], [75]. These modalities, however, are not utilized as commonly as radiation for the therapy of cancer [41]. Marini et al. [58] reported decreased expression of DR4 and increased expression of DR5 in colorectal cells receiving 10 Gy of radiation. Interestingly, DR5 expression was increased 18 h post-irradiation but not at 24 h post-irradiation. These studies evaluated both combined and sequential administration of radiation and TRAIL. Similar to this report, we observed increased expression of DR5 in our three cell lines (Fig. 5D). Interestingly, we also observed increased expression in DR4 in three of three colorectal cell lines examined (Fig. 5C), and no decreased expression. Though Marini et al. evaluated different cell lines than those evaluated here (Colo 205 and HCT-15), they did report enhanced killing when combined with radiation. In another study, radiation was reported to modulate sensitivity to TRAIL-induced apoptosis [59] with 5 Gy in HCT116 cells. The enhancement was low in this study, with 25% cell death with DR5 agonist (lexatumumab) alone enhanced to 40% when radiation was added. Notably, there was no enhancement when the DR4 agonist (mapatumumab) was used in combination with radiation in these same cells. There was no enhancement in TRAIL-induced apoptosis in combination with radiation in the other colorectal cell line (Colo 205) evaluated in the same study. Irradiation was performed immediately before administrating the antibodies, and it is likely that changes in gene expression are necessary to achieve maximum synergy between these two modalities. Here we report enhanced TRAIL receptor induced death following radiation in three of three colorectal cell lines (Fig. 7), including SW620 cells that are generally considered resistant to both Fas and TRAIL-induced cell death. This enhanced sensitivity to TRAIL-induced cell death was observable as long as 5 days post irradiation in these cells. In contrast, HCT116 cells, which were the most sensitive to TRAIL-induced cell death in the absence of radiation, lost some radiation-enhanced sensitivity to TRAIL 5-days post irradiation as compared to the sensitivity 3-days post irradiation (**Fig. 7C & 7F**). This sensitivity does not appear to be a result of the loss of radiation-induced changes in surface TRAIL receptors as changes in surface TRAIL were detectable as long as seven days following IR (**Fig. 6**). Overall, our data suggest that colorectal cells are more susceptible to TRAIL-induced death signals post-irradiation and that TRAIL receptor agonists, that signal both DR4 and DR5, are preferable.

As noted above, TNF-α and IFN-γ were reported to enhance TRAIL-induced cell death of tumor cells [41] and here we have shown that radiation can also enhance TRAIL-induced death, in the absence of such cytokines. Similarly, IFN-γ has been reported to enhance LT*β*R-induced cell death in tumor cells treated with the mAb (31G4D8) but was not essential for LT*β*R death induced by a pentameric form of the antibody (CBE11) [31]. We wanted to determine if tumor cell irradiation alone could enhance LT*β*R mediated cell death signaling in a manner similar to that observed with Fas and TRAIL killing, in the absence of exogenous cytokines. Interestingly, though the tumor cells utilized for these studies expressed high levels of surface LT*β*R (**Fig. 5A**), we were unable to induce cell death through this receptor pathway. Moreover, tumor cell irradiation did not sensitize cells to LT*β*R-induced cell death (**Fig. 8**), as it did for Fas and TRAIL-induced death. It remains to be determined, however, if irradiation could further enhance cell death of tumor cells treated with anti-LT*β*R mAb and IFN-γ in combination and this is currently under investigation. Additionally, it would be interesting to see if radiation can enhance tumor cell death triggered by the pentameric LT*β*R (which is not commercially available). Furthermore, while we observed increased expression of Fas, DR4, and DR5 in this study, we did not detect an increase in the expression of LTβR or TNF-R1 (Fig. 5A–B). These results indicate that LTβR agonists or TNF-R1 agonists may not be best suited for use in combination with radiation therapy. In fact, 10 Gy of radiation appears to mildly reduce expression of TNF-R1 in colorectal cells (Fig. 5B). However, further investigation is required to determine if there are conditions under which radiation can have an impact on the functional activity of these two pathways irrespective of changes in surface expression.

Marini et al. did report enhanced killing of two colorectal tumor cell lines at 12 and 48 h post-treatment. According to the authors of that study, TRAIL was added either directly after cell irradiation or 12 h later, to allow for receptor up-regulation. In our studies we irradiated tumor cells and waited several days post-irradiation before triggering the death receptors. Enhanced responses to both Fas and TRAIL receptor signaling were detected as long as five days after tumor cell irradiation (Fig. 4A–C and Fig. 7D–F). Moreover, we observed up-regulation of DR4 as early as 48 h post-irradiation and as long as seven days post-irradiation (Fig. 6). Overall, the results from Marini et al., in combination with the results of this current report provide evidence that the treatment window to benefit from radiation enhanced death receptor signaling can be as early as 12 h and as late as one week after radiation.

We showed that receptor-mediated apoptosis was enhanced by tumor cell irradiation (Fig. 9). Immune effector cells utilize these apoptotic pathways as a part of the death receptor killing mechanism. Killing initiated through Fas and TRAIL receptors are well-studied pathways that rely on induction of a cascade of apoptotic proteins, including caspases and Bcl-2 protein family members to execute death of a target tumor cell [28]. These pathways are also important for traditional granzyme/peforin mediated killing of tumor target cells. In particular, Bid is known to promote the intrinsic apoptotic pathway to amplify cell death. As such, cell death induced via death receptors can be modulated or prevented by high expression of intracellular sensitizers to apoptosis, such as Bcl-2, Bcl-X_L_, survivin, or cFLIP. Agents that can decrease the expression of these molecules have been reported to enhance death receptor mediated death. TNF-α and IFN-γ were shown to enhance TRAIL killing by decreasing bcl-xL expression [41]. We have also previously shown that bcl-2 mRNA expression was reduced following sub-lethal irradiation of tumor cells [35]. Huerta et al. [49] showed that SW620 cells have acquired genetic defects in apoptotic pathways, which may explain the ability of advanced cancer cells to escape anti-tumor immune cells. Here we show that bcl-2 family members cannot completely explain enhancement of death receptor killing (Fig. 10). For example, WiDr cells that were killed better by both Fas and TRAIL receptor signaling post-IR had the highest overall expression of Bcl-X_L_ and c-FLIP protein levels. SW620 cells displayed marked reduction in Bcl- X_L_ protein levels 72 h following 10 Gy IR; however, they were not sensitive to Fas-induced cell death at this time in contrast to HCT116 cells with no reduction that were very sensitive to Fas-induced cell death. Thus, enhanced killing of sub-lethally irradiated tumor cells did not correlate with changes in death receptor expression or other intracellular sensitizers to death receptor mediated apoptosis, such as c-FLIP or bcl-X_L_. We also found no correlation with changes in the expression of pro-apoptotic proteins, such as Bid, Puma, or Bax (Fig. 10G–I). We are currently exploring other pro-apoptotic and anti-apoptotic proteins that could be responsible for the observed differences in susceptibility. For example, White-Gilbertson [40] suggests that sensitivity to TRAIL in SW620 cells is regulated by ceramide production that modulates caspase-3 translocation to the nucleus.

Death receptor ligands, such as Fas-ligand and tumor necrosis factor-related apoptosis inducing ligand (TRAIL) are potentially useful for combination therapy of cancer. These death ligands can be administered as external soluble agents or, alternatively, by induction of antitumor CTL to attack target cells. It is important to note that the Fas Ligand (FasL) on immune cells are trimerized and commercially available recombinant FasL and Fas mAb (CH11) may not mimic the physiological signal. Administration of 10 Gy of radiation and a novel trimerized FasL protein, APO010, to HCT116 simultaneously resulted in enhanced DNA fragmentation [60]. SW620 cells, which are generally considered resistant to both FasL and TRAIL may be more susceptible to Fas signaling induced by APO010. That said, in vivo 10 Gy+APO010 every 48 h (begun the same day as IR) did not enhance the efficacy of radiation alone. This could be due to the insufficient circulating amounts of this antibody, poor half-life, or inability of the antibody to access modulated tumors. In contrast, induction of anti-tumor CTLs or NK cells may be able to better suited to utilize this pathway. Future investigations will evaluate combination RT immunotherapy experiments evaluating human tumor cell lines in an in vivo murine xenograft model.

Antitumor effector cells occur naturally in cancer patients and can be generated or increased by numerous therapeutic approaches [19], [21], [22]. We are interested in exploring basic aspects of the death receptor pathways utilized by cytolytic immune cells, which may have implications for designing improved immunotherapeutic strategies. In this study we systematically evaluate surface expression, as well as functional enhancement, of common receptors used to induce death in tumor cells following sub-lethal tumor cell irradiation. Overall, we found that sub-lethal irradiation of human colorectal tumor cells imparts enhanced and sustained susceptibility to multiple death signaling receptor pathways. Our results indicate that utilizing modalities, such as ionizing radiation, which is commonly used in the clinic, may promote more favorable immune cell activity toward colorectal tumor cells. The mechanism by which radiation sensitizes colorectal carcinoma cells to killing through Fas and TRAIL, and the potential for clinical application are currently being further explored.

## Materials and Methods

### Cell lines

Colorectal tumor cell lines HCT116 and WiDr were generously provided from the Laboratory of Tumor Immunology and Biology, NCI, NIH [34]. Human colorectal carcinoma cell line SW620 was generously provided by Zhi-Ren Liu [76] from Georgia State University, Department of Biology. HT-29 cells were originally purchased from ATCC. All cells were cultured in media designated by ATCC for propagation and maintenance. Cells were incubated at 37°C incubator with 5% CO_2_.

### Tumor irradiation

Human tumor cells were harvested while in log-growth phase. Tumor cells, in 10 ml suspension, were placed on ice and irradiated (0–15 Gy) by ^137^Cs source (Gammacell-1000; AECL/Nordion. Kanata, Ontario, Canada) at a dose rate of 0.70 Gy/min. Control samples were also placed on ice but not irradiated. Both irradiated and non-irradiated cells were then washed in fresh media and seeded in tissue culture flasks.

### Tumor cell proliferation assay

Proliferation rates were determined by measuring the uptake of 5-bromo-2″deoxyuridine (BRDU) in triplicate. Carcinoma cell lines were treated with 0, 2.5, 5, or 10 Gy of radiation, and 1×10^4^ cells were plated in 50 ul and were allowed to adhere overnight in 10% media in a 96-well plate. After three days, plates were harvested and assayed for BRDU incorporation 18 h before harvest BRDU was added to the culture. Proliferation was measured using the DELFIA cell proliferation kit, according to manufacturer's instructions, and luminescence was detected using a Victor 3 plate reader (Perkin Elmer. Waltham, Massachusetts).

### Flow cytometric analysis

Cell surface staining of tumor cells was performed using the following primary labeled mAb - CD95-PE, LTβR-PE, DR4-PE, DR-5-APC - and the appropriate isotype matched controls. Surface expression of TNF-R1 (CD120a) was performed with a two-step protocol using biotin labeled TNF-R1, followed by streptavidin-PE secondary staining (BD Biosciences. San Diego, CA). 7AAD staining was used as a measure of cell death, following manufacturer's instructions. Antibodies were purchased from either BD Biosciences (San Diego, CA) or BioLegend (San Diego, CA). Stained cells were acquired on a BD Fortessa flow cytometer using FACSDiva software (BD PharMingen. San Diego, CA). Isotype control staining was less than 5% for all samples analyzed. Dead cells were excluded from the analysis based on scatter profile.

### Functional death receptor assay

Human tumor cells were non-irradiated (0 Gy) or irradiated (2.5, 5, and 10 Gy) and re-cultured. After 72 h or 120 h, cells were harvested and counted. Human tumor cells were incubated for 3 h with varying concentrations of agonistic anti-Fas antibody, clone CH11 (MBL. Watertown, MA) or recombinant TRAIL protein (Millipore. Billerica, MA). Control cells were incubated with IgM isotype control antibody (BD Biosciences. San Diego, CA). Jurkat cells were used as a positive control for Fas-mediated cytotoxicity. Cells were subsequently fixed and permeabilized before being stained for intracellular active caspase-3 with a PE-labeled monoclonal antibody (BD Biosciences. San Diego, CA). The level of activated caspase-3 was quantified by flow cytometry, as described above. Alternatively, for experiments to confirm apoptotic cell death, cells were harvested and stained using Annexin-V-PE and 7-actinomycin D (7-AAD) according to manufacturers instructions (BD Biosciences. San Diego, CA). Background staining observed with IgM isotype control antibody (Fas functional assays) and cells incubated in media (for TRAIL functional assays) was subtracted out.

For LT*β*R death assays, 72 hr post-IR cells were loaded in culture plates pre-coated with 10 µg/mL or 1 µg/mL of agonistic anti-LT*β*R monoclonal antibody (mAb) in conjunction with 50 units/ml IFN-γ (for HT29 cells) or media (SW620, WiDr, HCT116) and were incubated for 48–72 h. Cell viability was determined using the DNA-specific viability dye 7-AAD comparing treated cells versus cells cultured in medium alone and isotype control mAb. Background staining observed with mouse IgG2b isotype control antibody was subtracted out.

### Western blotting

Antibodies used in this study were directed against the following: c-FLIP (Cell Signaling #3210. Danvers, MA) at 1∶1000, Bcl-X_L_ (Cell Signaling #2764) at 1∶1000, Bid (Cell Signaling #2002. Danvers, MA) at 1∶1000, Puma (Cell Signaling #4976. Danvers, MA) at 1∶1000, Bax (Cell Signaling #5023. Danvers, MA) at 1∶1000 and β-actin (Cell Signaling rabbit #4970, mouse #3700. Danvers, MA). Human colorectal cancer cells were treated with ionizing radiation at doses ranging from 0 Gy to 10 Gy. Cells were lysed 72 h post-irradiation using NP40 cell lysis buffer. Protein concentration of lysates was determined by a BCA Protein Assay (Thermo Scientific Pierce. Rockford, IL) and 7 µg of protein used in Western blot analysis. Samples for electrophoresis were prepared with Laemmli sample buffer (BIO-RAD. Hercules, CA), 5% β-mercaptoethanol, and were run on 12% SDS-PAGE gels (BIO-RAD. Hercules, CA). Proteins were transferred onto nitrocellulose membranes for 1 h at 100 V in a mini-Protean III transfer tank (BIO-RAD. Hercules, CA), and blocked for 1 h at room temperature in blocking buffer 1× TBS, 0.1% Tween-20 (TBS-T) with 5% w/v nonfat dry milk. Membranes were incubated overnight at 4°C, with antibodies in primary antibody dilution buffer containing 1× TBS, 0.1% Tween-20 with 5% BSA. Membranes were washed 3× for 30 min in TBS-T, and incubated with HRP-conjugated anti-rabbit antibody (GE Healthcare. Piscataway, NJ) at room temperature for 1 h. Membranes were subsequently washed with TBS-T and developed with Amersham ECL Western blotting analysis system (GE Healthcare. Piscataway, NJ). Following detection of cFLIP and Bcl-X_L_ proteins, membranes were striped and re-probed for β-actin protein.

### RNA isolation

Cells were irradiated and seeded in T-75 flasks at 0.5 to 1×10^7^ cells/flask. After 24 h, cells were harvested from flasks and total RNA was extracted and purified using the RNeasy mini kit (Qiagen Inc. Valencia, CA) according to the manufacturer's instructions. Purified RNA was DNase-treated by Rnase-free DNase (Qiagen. Valencia, CA) according to the manufacturer's instructions.

### Quantitative real-time PCR

For selected cell lines, real-time PCR analysis was performed on cDNA isolated from irradiated or non-irradiated cells. cDNA was synthesized from 500 ng RNA isolated in a standard RT reaction. Cell lines were subjected to radiation at doses between 0 Gy to 10 Gy and harvested 24, 48, and 72 h post-irradiation. RNA samples were reverse transcribed to cDNA using random hexamer primers (Finnzymes. Vantaa, Finland). Amplification of cDNA was done using DyNaAmo SYBR Green (Finnzymes. Vantaa, Finland) and the following sequences of primer sets: cFLIP (l) forward 5′ CGG ACT ATA GAG TGC TGA TGG 3′ and reverse 5′ GAT TAT CAG GCA GAT TCC TAG 3′; cFLIP (s) forward 5′ CGG ACT ATA GAG TGC TGA TGG 3′ and reverse 5′ AGA TCA GGA CAA TGG GCA TAG 3′; GAPDH forward 5′ TTC GTC ATG GGT GTG AAC 3′ and reverse 5′ AGT GAG CTT CCC GTT CAG C 3′; Bcl-2 forward 5′ GAG GAT TGT GGC CTT CTT TG 3′ and reverse 5′ GCC GGT TCA GGT ACT CAG TC 3′; survivin forward 5′ CCA GAT GAC GAC CCC ATA GAG 3′ and reverse 5′ TTG TTG GTT TCC TTT GCA ATT TT 3′; HPRT forward 5′ TGG ACA GGA CTG AAC GTC TTG 3′ and reverse 5′ CCA GCA GGT CAG CAA AGA ATT TA 3′. Primers were synthesized by Integrated DNA technologies (Coralville, IA). Cycling conditions were set as the following: (a) 2 min at 5°C (b) 15 min at 95°C (c) 30 cycles of: 15 s at 95°C, 30 s at (57°C for bcl-2/GAPDH, 60°C for cFLIP/survivin/HPRT), 30 s at 72°C, followed by (d) 7 min at 72°C.

### Statistical Analysis

Tests of significance are reported as p values, derived from Student's t-test, using a 2-tailed distribution and calculated at 95% confidence. Statistical tests were carried out with the software packages Graph-Pad InStat/Prism (GraphPad Software, Inc. San Diego, CA).
